# Supplementary material for: Allosteric Regulation of RNA Affinity by Motif V-VI Coupling in West Nile Virus NS3 Helicase
Source: bioRxiv. 2025 Jul 4:2025.07.04.663198. Preprint. [Version 1] doi: 10.1101/2025.07.04.663198 (PMC12236514; doi:10.1101/2025.07.04.663198)

Supporting Information for: Allosteric Regulation of RNA Affinity by Motif V-VI Coupling in West Nile Virus NS3 Helicase

Priti Roy and Martin McCullagh*

Department of Chemistry, Oklahoma State University, Stillwater, OK, 74074, USA

E-mail: martin.mccullagh@okstate.edu

Table S1: Occurrence probability of conserved RNA contacts in the ssRNA state of WNV NS3h mutants. Wild-type data is included for comparison. A 5 Å of cut-off distance is used between atoms of protein and ssRNA.

**Motif**	**Residue**	**Wild-type**	**D471E**	**D471L**
**Ia**	P224	99%	97.73%	100%
**Ia**	T225	99%	98.18%	100%
**Ia**	R226	99%	99.99%	100%
**II**	T290	64%	0.15%	68.27%
**II**	D291	99%	59.93%	97.98%
**IV**	S365	35%	99.85%	97.51%
**IVa**	R388	99%	99.96%	100%
**V**	D410	97%	7.64%	100%

Table S2: Occurrence probability of conserved RNA contacts in the ssRNA+ATP state of WNV NS3h mutants. Wild-type data is included for comparison. A 5 Å of cut-off distance is used between atoms of protein and ssRNA.

**Motif**	**Residue**	**Wild-type**	**D471E**	**D471L**
**Ia**	P224	99%	99.76%	98.47%
**Ia**	T225	81%	99.65%	99.86%
**Ia**	R226	99%	100%	100%
**II**	T290	8%	14.14%	23%%
**II**	D291	99%	99.27%	94.05%
**IV**	P364	93%	80.03%	99.52%
**IV**	S365	99%	75.84%	97.81%
**IV**	V366	98%	73.35%	96%
**IV**	K367	56%	72.53%	40.6%
**IVa**	S387	99%	1.31%	96.79%
**IVa**	R388	99%	99.99%	100%
**IVa**	K389	63%	14.12%	32.19%
**V**	T409	99%	88.62%	100%
**V**	D410	99%	27.62%	99.98%
**V**	I411	99%	99.58%	100%

Table S3: Occurrence probability of conserved RNA contacts in the ssRNA+ADP+P_i state of WNV NS3h mutants. Wild-type data is included for comparison. A 5 Å of cut-off distance is used between atoms of protein and ssRNA.

Motif	Residue	Wild-type	D471E	D471L
**Ia**	P224	99%	69.91%	99.03%
**Ia**	T225	99%	91.47%	99.94%
**Ia**	R226	99%	100%	100%
**II**	D291	99%	80.28%	99.75%
**IV**	S365	97%	99.69%	36.72%
**IV**	V366	99%	97.4%	99.24%
**IV**	K367	84%	14.07%	4.95%
**IVa**	S387	99%	99.83%	99.67%
**IVa**	R388	99%	100%	99.88%
**IVa**	K389	93%	11.81%	39.04%
**V**	T409	99%	100%	99.99%
**V**	D410	99%	57.53%	85.74%
**V**	I411	99%	100%	100%

Table S4: Occurrence probability of conserved RNA contacts in the ssRNA+ADP state of WNV NS3h mutants. Wild-type data is included for comparison. A 5 Å of cut-off distance is used between atoms of protein and ssRNA.

Motif	Residue	Wild-type	D471E	D471L
**Ia**	P224	99%	100%	100%
**Ia**	T225	99%	100%	100%
**Ia**	R226	99%	100%	100%
**II**	T290	63%	65.40%	1.12%
**II**	D291	99%	99.68	92.65%
**IV**	P364	99%	100%	76.63%
**IV**	S365	99%	98.96%	23.83%
**IV**	V366	99%	98.02%	24.64%
**IV**	K367	95%	62.02%	7.92%
**IVa**	R388	99%	99.99%	100%
**V**	T409	98%	100%	100%
**V**	I411	98%	99.99%	100%

Table S5: Occurrence probability of conserved ATP contacts in the ssRNA+ATP state of WNV NS3h mutants. Wild-type data is included for compariso. A 5 Å of cut-off distance is used between atoms of protein and ATP.

**Motif**	**Residue**	**Wild-type**	**D471E**	**D471L**
**I**	H195	61%	0.25%	100%
**I**	P196	99%	22.55%	100%
**I**	G197	99%	99.75%	100%
**I**	A198	99%	100%	100%
**I**	G199	99%	100%	100%
**I**	K200	99%	100%	100%
**I**	T201	99%	100%	100%
**V**	N417	92%	97.45%	70.73%
**VI**	R459	99%	0.01%	–
**VI**	R462	99%	21.58%	–

Table S6: Occurrence probability of conserved ADP+P_i contacts in the ssRNA+ADP+P_i state of WNV NS3h mutants. Wild-type data is included for compariso. A 5 Å of cut-off distance is used between atoms of protein and ADP+P_i.

Motif	Residue	Wild-type	D471E	D471L
**I**	H195	86%	99.9%	1.82%
**I**	P196	99%	99.85%	75.26%
**I**	G197	99%	100%	33.40%
**I**	A198	99%	100%	18.48%
**I**	G199	99%	100%	92.80%
**I**	K200	99%	100%	90.54%
**I**	T201	99%	100%	100%
**II**	E286	98%	96.55%	–
**V**	G415	99%	0.11%	50.47%
**V**	N417	90%	32.13%	97.9%
**VI**	Q455	98%	–	–
**VI**	R459	99%	–	–
**VI**	R462	99%	0.18%	35.66%
**VI**	N465	99%	2.42%	34.09%

Table S7: Occurrence probability of conserved ADP contacts in the ssRNA+ADP state of WNV NS3h mutants. Wild-type data is included for compariso. A 5 Å of cut-off distance is used between atoms of protein and ADP.

Motif	Residue	Wild-type	D471E	D471L
**I**	P196	97%	86.66%	99.85%
**I**	G197	99%	91.77%	100%
**I**	A198	99%	99.96%	100%
**I**	G199	99%	100%	100%
**I**	K200	99%	100%	100%
**I**	T201	99%	100%	100%
**II**	E286	68%	5.83%	1.28%
**V**	G415	23%	78.21%	1.64%
**V**	N417	94%	77.42%	74.88%
**VI**	R459	97%	0.54%	0.59%
**VI**	R461	70%	0.02%	0.37
**VI**	N465	97%	9.7%	77.38%

Table S8: Probability of oxygen atoms participated in the octahedral coordination of Mg^{2+} in WNV NS3H mutant. This calculation is performed for all hydrolysis states of D471L. The octahedral shell is defined by oxygen atoms with 2.5 Å of Mg^{2+}.

Atom Name	ssRNA+ATP	ssRNA+ADP+P_i	ssRNA+ADP
$OG1_{T200}$	99.97%	99.63%	99.84%
$O2G_{ATP}$	100%	–	–
$O1B_{ATP}$	100%	–	–
$O1B_{ADP}$	–	100%	100%
$O3_{P_i}$	–	100%	–
O_{W1}	100%	100%	100%
O_{W2}	100%	100%	100%
O_{W3}	100%	100%	100%
O_{W4}	–	–	100%

Figure S1: **2D histogram of backbone dihedral angles of the entire NS3h** The ϕ and ψ are denoted as backbone dihedrals and these values are calculated for each hydrolysis state of Wild-type and NS3h mutants. Units in degrees.

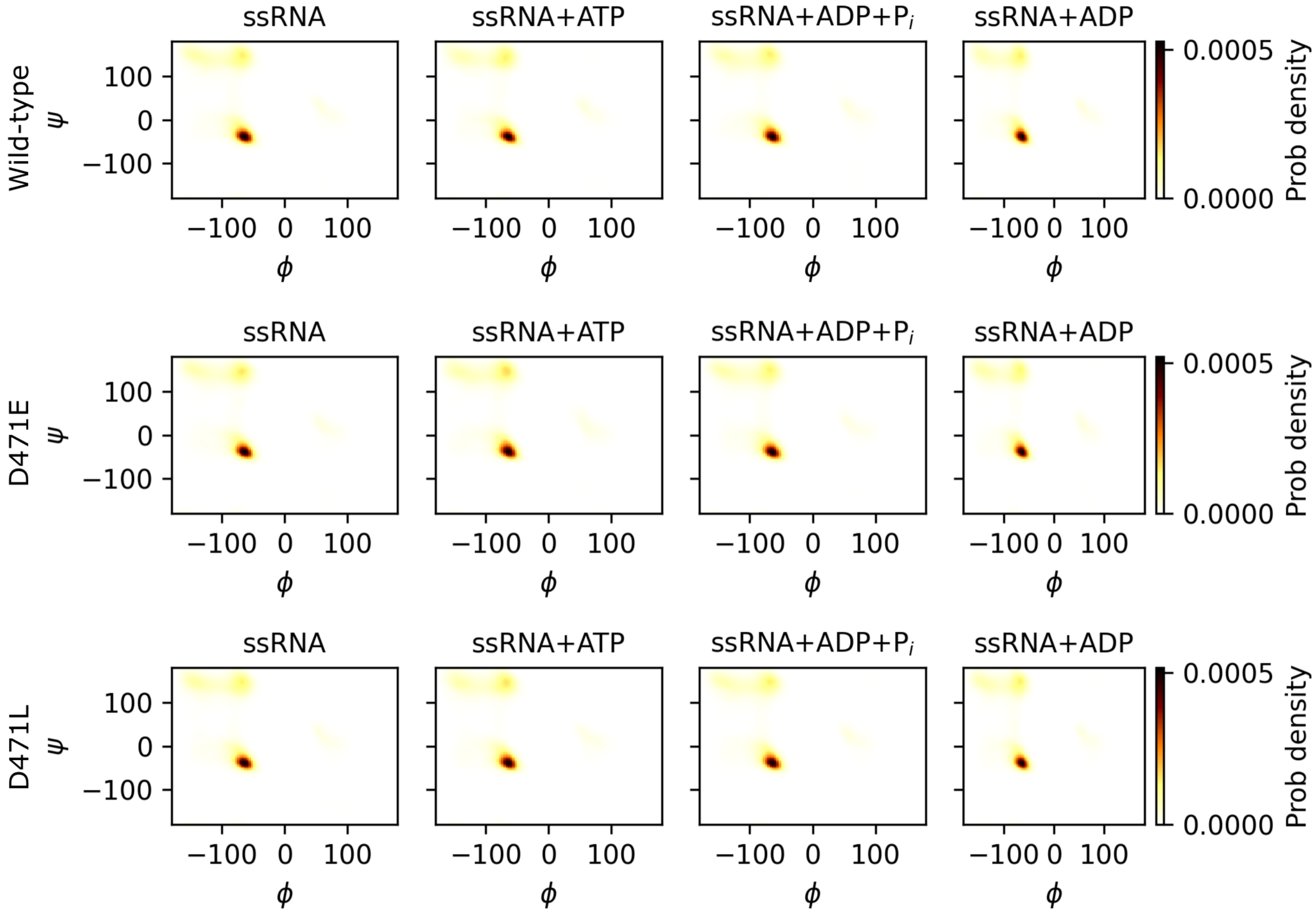

Figure S2: **ATP-dependent correlation between nucleotide and RNA affinity in WNV NS3h D471E.** (A–C) Residue-wise cross-correlation plots comparing nucleotide (ADP) and RNA(P) binding affinities for three hydrolysis states: (A) ssRNA+ATP (pre-hydrolysis), (B) ssRNA+ADP+P_i (post-hydrolysis I), and (C) ssRNA+ADP (post-hydrolysis II). The black rectangle in each plot denote significant correlation region observed in Wild-type. For analysis details see main text.

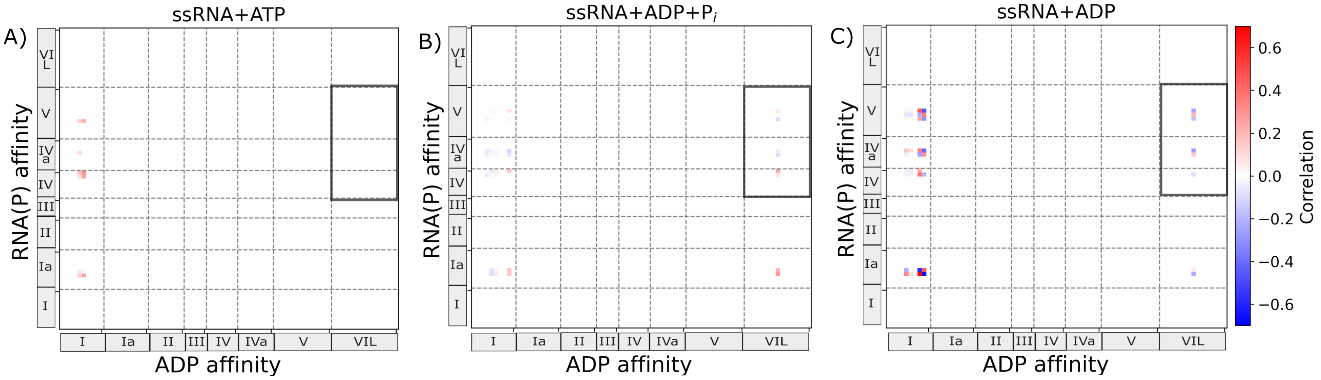

Figure S3: **ATP-dependent correlation between nucleotide and RNA affinity in WNV NS3h D471L.** (A–C) Residue-wise cross-correlation plots comparing nucleotide (ADP) and RNA(P) binding affinities for three hydrolysis states: (A) ssRNA+ATP (pre-hydrolysis), (B) ssRNA+ADP+P_i (post-hydrolysis I), and (C) ssRNA+ADP (post-hydrolysis II). The black rectangle in each plot denote significant correlation region observed in Wild-type. For analysis details see main text.

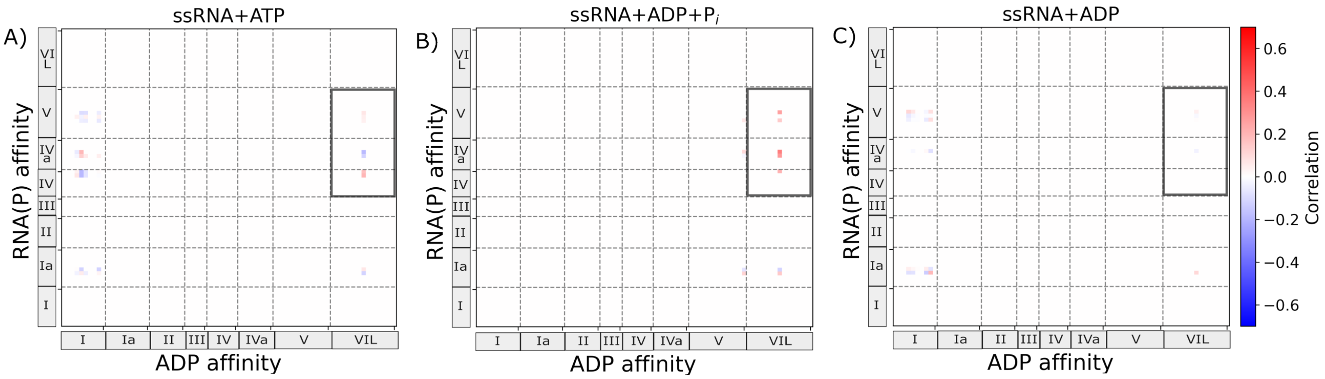

Figure S4: **Residue level phosphate affinity change at RNA-cleft motifs Ia, IV, IVa and V in Wild-type**. The binding affinity change for each protein residue for phosphates P_1 to P_6 are presented from top to bottom panel. The deviation correspond to difference of the ensemble averages, those are computed from entire NPT ensemble of the respective system. The errorbar represents propagated standard deviation of two compared systems where standard deviations are computed from the averages of 50k frames chunks. See main text for details. The units in kcal.mol^{-1}

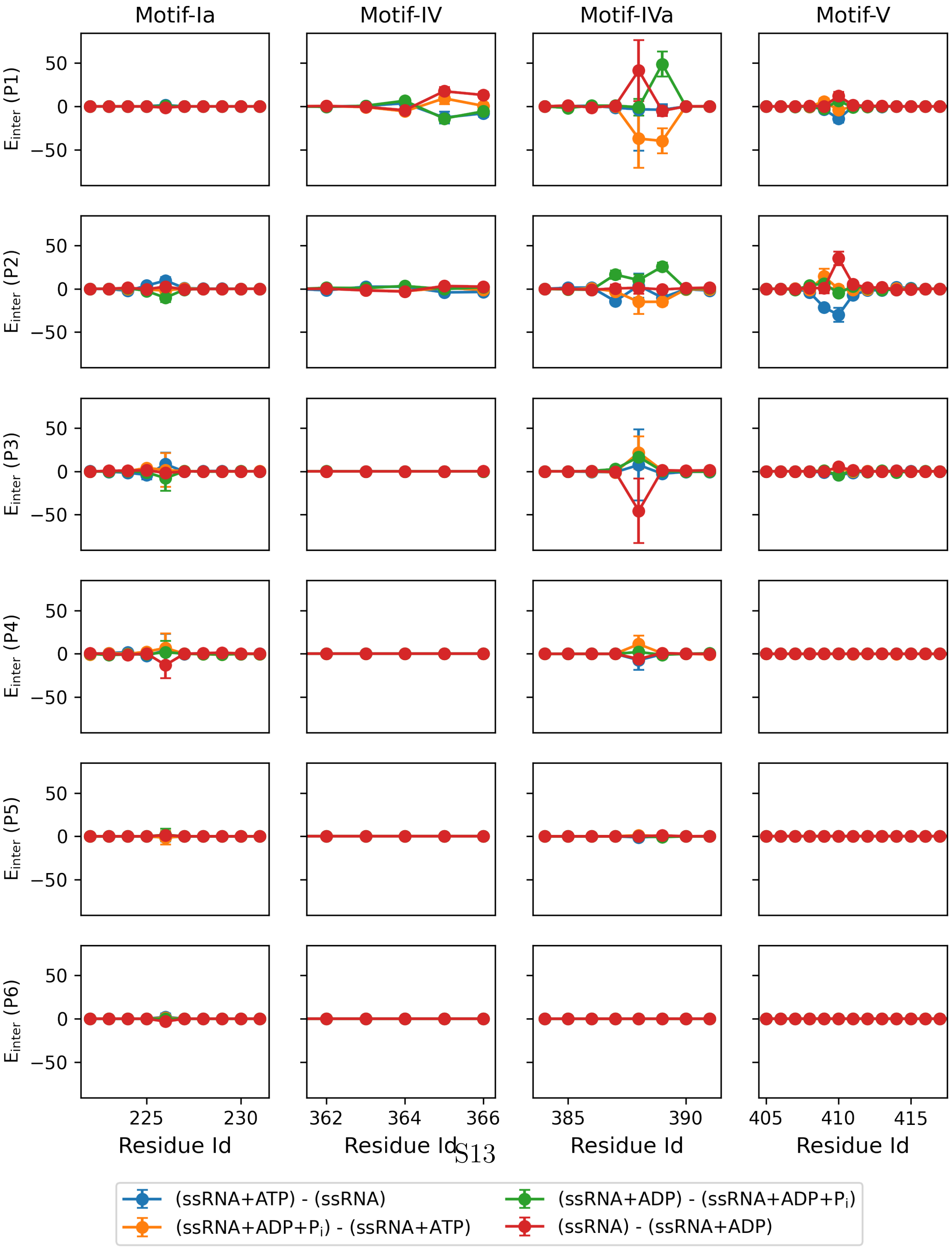

Figure S5: **ATP-dependent ssRNA phosphate affinity change for motif IVa and V in the D471L**. Residue wise differential binding enthalpy (ΔE_{inter}) of motif **V** for **P2**, motif **IVa** for **P1** and motif **IVa** for **P3** shown in left, middle and right columns respectively. The deviation corresponds to difference of the averages, those are computed from entire NPT ensemble of the respective states. The error bar represents propagated standard deviation of two compared states where standard deviations are computed from the averages of 50k frames chunks. (A-C) These plots mark the change in affinity while protein transition from ssRNA to ssRNA+ATP state. (D-E) Changes in RNA phosphate affinity after protein moving from ssRNA+ATP state to ssRNA+ADP+P_i state. (G-I) RNA phosphate affinity change due to transition of protein from ssRNA+ADP+P_i to ssRNA+ADP state. (J-L) Changes in RNA phosphate affinity while protein move from ssRNA+ADP state of previous cycle to the ssRNA state of next cycle. Units of ΔE_{inter} is in kcal·mol^{-1}

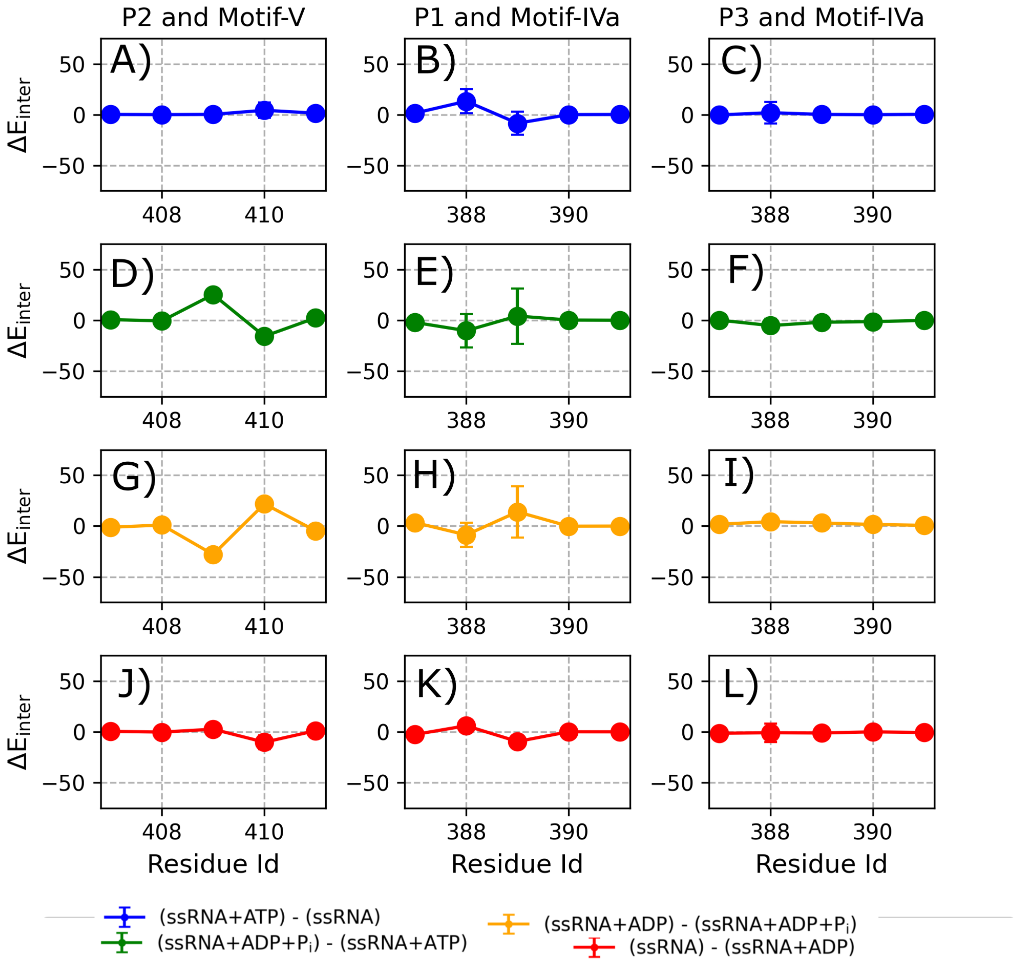

Figure S6: **Correlation of ADP affinity of motif VIL and key wild-type RNA contacts in D471L** The RNA interacting key residues were identified from ATP-dependent changes in the protein residue and RNA phosphates affinity (Figure **??**) in the wild-type. The key interacting residue pairs are: P2 and T409 of motif **V**, P2-D410 of motif **V**, P1 and R388 from motif **IVa**, P1 and K389 from motif **IVa**, P3 and R388 from motif **IVa**, P3 and K389 from motif **IVa**. Correlation between the interaction energy of these pairs in the RNA-cleft and ADP interaction energy of motif **VIL** calculated for hydrolysis states ATP, ADP+P_i and ADP.

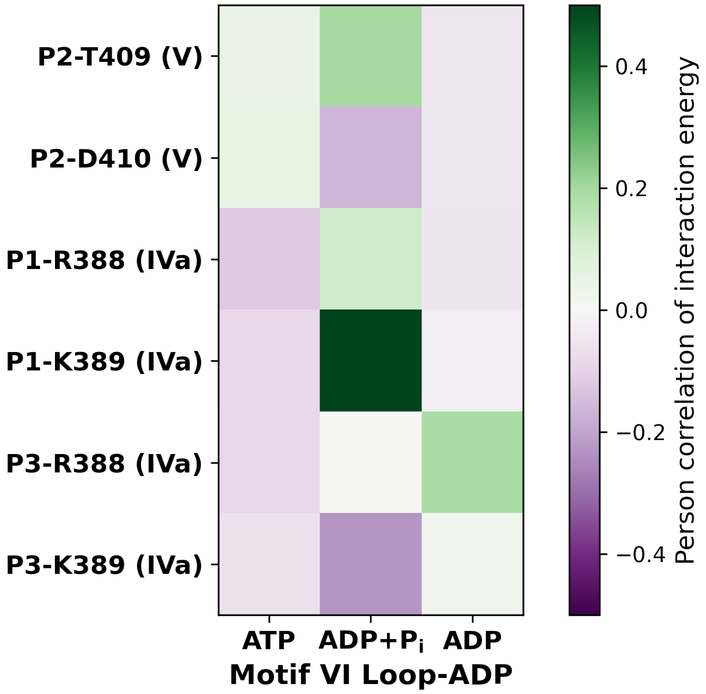

Figure S7: **Time evolution of distances of residue pairs between motif V and VIL**. The plots represent the center-of-mass distances for backbones or sidechains for residue pairs showed correlation coefficient equal or greater than 0.55 in the hydrolysis states (see main text). These time traces demonstrate the stability or fluctuation along with spatial proximity of the motif V and VI residue pairs. The distance units in Å.

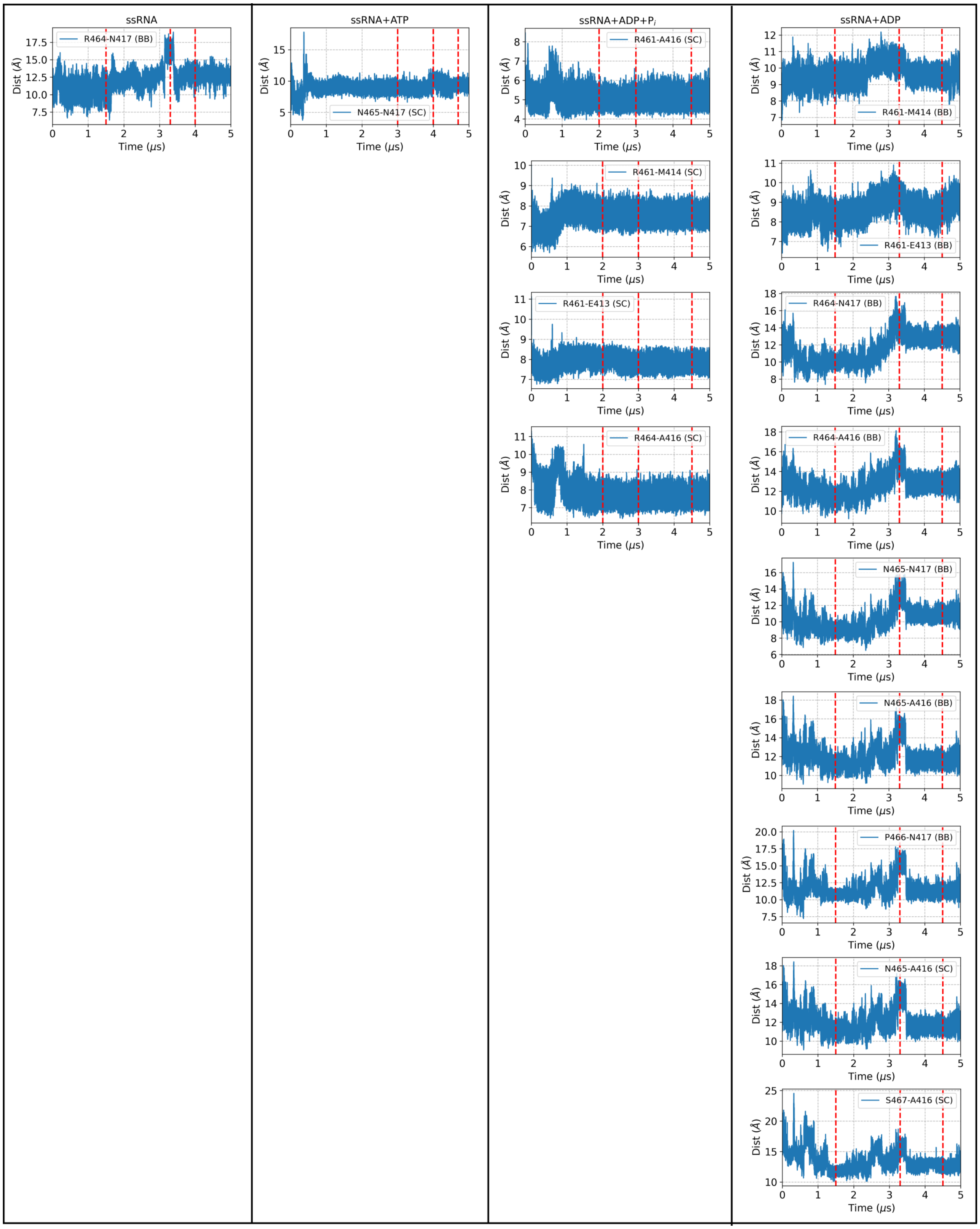

Figure S8: **Sampling of E413-R461 hydrogen bond in the Wild-type ssRNA and ssRNA+ATP states.** The backbone and sidechain atoms of E413 act as an acceptor while the sidechain atoms of R461 act as a donor.

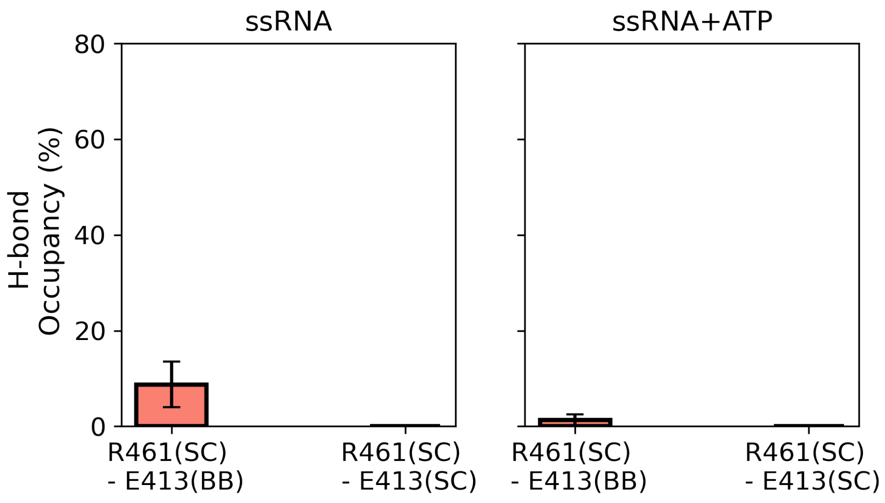

Figure S9: **Sampling of E413-R461 hydrogen bond in the hydrolysis states of D471L.** The backbone and sidechain atoms of E413 act as an acceptor while the sidechain atoms of R461 act as a donor.

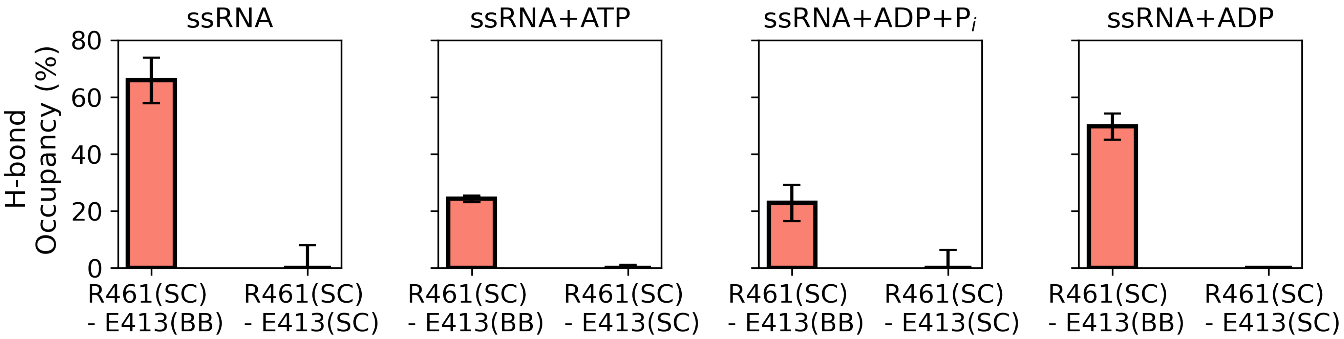

Figure S10: **Structural sampling of 3_{10}-helix of motif V in the hydrolysis states of D471L.** These 2D free energy plots show ATP-dependent sampling of ϕ and ψ angles of 3_{10}-helix. Contour lines are plotted with an interval of 0.5 $k_\beta T$, ranging from 0 to 4 $k_\beta T$.

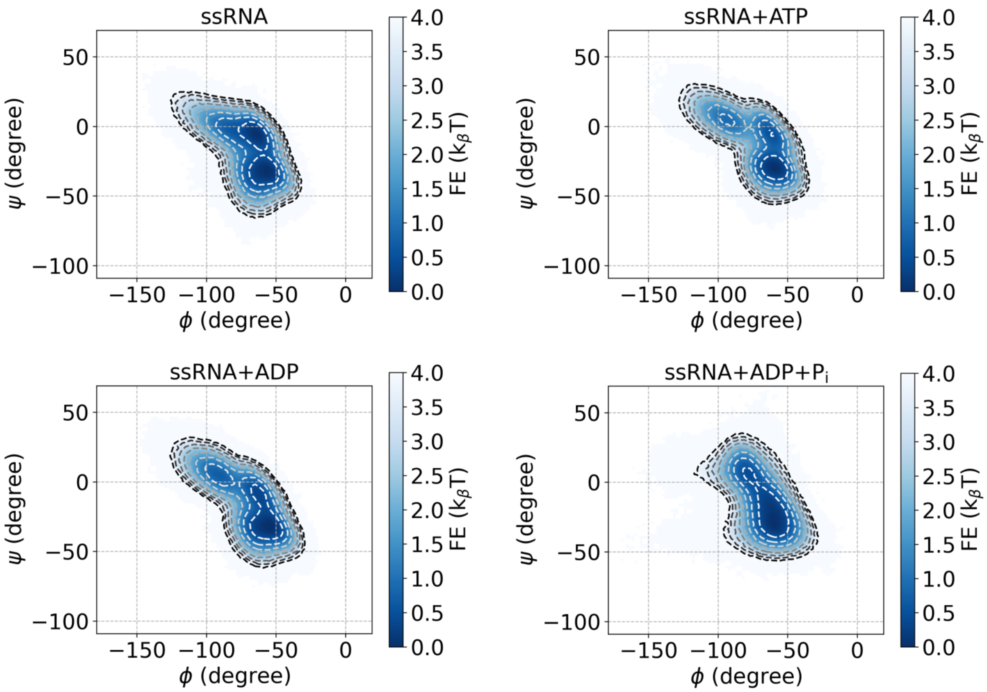

Supplement: Supplement 1 [file media-1.pdf]
